# The RNA helicase DDX3 promotes *IFNB* transcription via enhancing IRF-3/p300 holocomplex binding to the *IFNB* promoter

**DOI:** 10.1038/s41598-022-07876-z

**Published:** 2022-03-10

**Authors:** Wilaiporn Saikruang, Lena Ang Yan Ping, Hiroto Abe, Dacquin M. Kasumba, Hiroki Kato, Takashi Fujita

**Affiliations:** 1 Division of Integrated Life Science, Graduate School of Biostudies, Kyoto University, Kyoto, 606-8501 Japan; 2 Laboratory of Regulatory Information, Institute for Frontier Life and Medical Sciences, Kyoto University, 606-8507 Kyoto, Japan; 3 Institute for Cardiovascular Immunology, University Hospital Bonn, University of Bonn, 53127 Bonn, Germany

**Keywords:** Cell biology, Immunology, Molecular biology

## Abstract

The human DEAD-box protein 3 (DDX3) has been reported as a positive regulator and functions in the induction of type I interferon signaling. We elucidated the function of DDX3 in the positive regulation of IFNB production in non-pDC cells. We found that DDX3 regulates virus-induced activation of *IFNB* at the level of IRF-3. However, it does not affect conventional innate signaling, including IRF-3 phosphorylation, dimerization, or nuclear translocation of IRF-3, but has some downstream events after IRF-3 phosphorylation. Co-immunoprecipitation analyses revealed that DDX3 interacts with IRF-3 through its DNA-binding domain and promotes IRF-3-mediated *IFNB* promoter activation. DDX3 does not affect the formation of the IRF-3/p300/CBP complex. Instead, ChIP and EMSA assay revealed that DDX3 promotes the recruitment of IRF-3 and transcriptional co-activator p300/CBP to the *IFNB* promoter. The ATP binding pocket of DDX3 is involved in this association and is essential for the transcriptional activation. Taken together, our study demonstrates that DDX3 plays an important role in guiding a transcription factor complex formed by antiviral signaling to the target gene promoter.

## Introduction

Innate immunity is the first line of defense against invading pathogens. Host cells recognize the pathogen-associated molecular patterns (PAMPs) by pathogen recognition receptors (PRRs), including Toll-like receptors (TLRs), retinoic acid-inducible gene I (RIG-I)-like receptors (RLRs), nucleotide-binding oligomerization domain (NOD)-like receptors (NLRs), and intracellular DNA sensors, and initiate antiviral responses^1^. Viral double-stranded RNA (dsRNA) or bacterial lipopolysaccharide (LPS) in the endosome is recognized by TLR3 or TLR4, respectively, whereas cytoplasmic viral dsRNA is sensed by RIG-I, MDA5, or protein kinase R (PKR)^2–5^.

Upon sensing cytoplasmic viral RNAs, N-terminal caspase activation and recruitment domain (CARD) of RIG-I and MDA5 interact with mitochondrial signaling adapter (MAVS), which subsequently recruit the downstream proteins kinases TBK1 or IKKε, which then phosphorylate and activate IRF-3^6^. IRF-3 is the most important factor in the regulation of virus-induced interferon (IFN) gene activation. IRF-3 is usually found in the cytoplasm in an inactive form^7^. Upon virus infection, IRF-3 is activated to promote antiviral responses. Activation of IRF-3 involves a cascade of events: phosphorylation at Ser 386, homodimer formation, nuclear translocation, and assembly of a complex containing p300/CBP on the regulatory elements of type I IFN promoter to turn on transcription^7–12^. Secreted IFN binds to the IFN receptor and activates the JAK/STAT signaling pathway to induce the expression of interferon-stimulated genes (ISGs), which play an essential role in antiviral activity^13,14^.

DDX3 is a member of the DEAD (Asp-Glu-Ala-Asp)-box helicase family^15,16^. DDX3 exists in two isoforms, DDX3X and DDX3Y, located on the X and Y chromosomes, respectively^17,18^. DDX3X (referred to as DDX3) is ubiquitously expressed in most tissues and cells, whereas the expression of DDX3Y is limited to the male germline^19^. DDX3X and DDX3Y were reported to have partial redundant functions^20^. Like most other DEAD-box helicases, DDX3 is a multifunctional protein with functions in RNA metabolism, including RNA transcription, RNA splicing, RNA transport, RNA degradation, and translation^21,22^.

There have been several reports claiming that DDX3 is involved in virus-induced signaling leading to IFN production. It was reported that DDX3 recognizes viral RNA and associates with MAVS, suggesting that DDX3 itself acts as a viral RNA sensor upstream of MAVS and TBK1/IKKε^23^. DDX3 was identified as a target of Vaccinia virus immune evading factor K7 and interacts with IKKε to facilitate IRF-3 activation^24^. Interaction of DDX3 with IKKε results in the phosphorylation of DDX3 and facilitates IRF-3 phosphorylation by IKKε, and DDX3 acts as an essential scaffold for the kinase/substrate complex^25^. Another report identified DDX3 as an interacting protein of TBK1 and like IKKε, TBK1 phosphorylates DDX3^26^. Although the precise mechanism has not been delineated, it was demonstrated that DDX3 is recruited to the type I IFN promoter by ChIP analysis^26^. The above results were obtained using non-pDC cells. pDC is a major IFN-α producer that utilizes distinct sensor TLR7/9, and kinases NIK, IKKα, and IRF-7. DDX3 facilitates this pathway to activate IRF-7^27^; however, the biological functions of DDX3 in innate antiviral immunity are not fully understood.

We elucidated the function of DDX3 in the positive regulation of IFN-β production in non-pDC cells. We determined that DDX3 regulates virus-induced activation of type I IFN at the level of IRF-3. However, our study suggests that DDX3 is not involved in the phosphorylation, dimerization, or nuclear translocation of IRF-3, but has some downstream events after IRF-3 phosphorylation. Co-immunoprecipitation analyses demonstrated that DDX3 has intrinsic activity to interact with IRF-3 and promotes IRF-3-mediated *IFNB* promoter activation. DDX3 interacts with IRF-3 through its DNA-binding domain. However, it does not affect the formation of the IRF-3/p300/CBP complex. Instead, DDX3 promotes the recruitment of IRF-3 and transcriptional co-activator p300/CBP to its target promoter site. In addition, we found that DDX3 promotes IRF-3-mediated *IFNB* promoter activation and augments IFN-β production in response to viral infection through its interaction with IRF-3.

## Results

### DDX3 positively regulates the antiviral innate immune responses

Previous studies reported that DDX3 positively regulates RLR signaling to facilitate type I IFN production^23–26,28^. As these reports suggested that DDX3 acts as multifunctional adaptor molecule in the RLR signaling pathway, we re-investigated its role in RLR signaling. To further substantiate the biological role of DDX3 in the innate antiviral response, we generated DDX3-deficient 293T and HeLa cells by CRISPR/Cas9 gene editing. Both DDX3-deficient 293T and HeLa cells contain non-functional N-terminally truncated proteins (Supplementary Fig. 1a).

**Figure 1:**
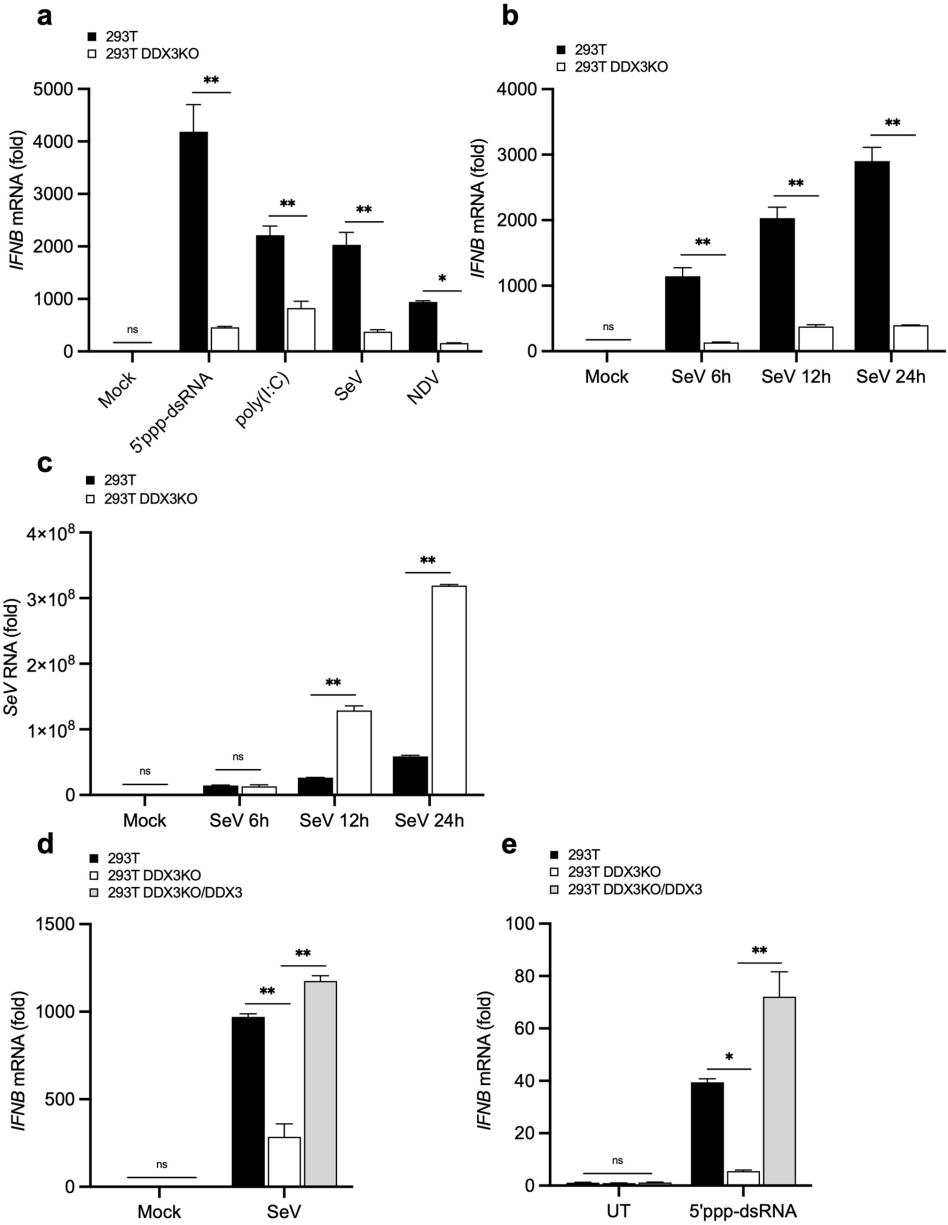
Effects of DDX3 KO on *IFNB* gene expression stimulated by viral infection or dsRNA. (**a**) q-PCR analysis of *IFNB* mRNA in 293T and 293T DDX3KO cells transfected with 5′ppp-dsRNA or HMW poly (I:C) or infected with SeV or NDV for 12 h. The results are presented as fold expression of *IFNB* mRNA to that of *GAPDH* mRNA. (**b**) q-PCR analysis of *IFNB* mRNA in 293T and 293T DDX3KO cells infected with SeV for indicated times. (**c**) q-PCR analysis of *SeV* RNA in 293T and 293T DDX3KO cells infected with SeV for indicated times. (**d**) q-PCR analysis of *IFNB* mRNA in 293T, 293T DDX3KO, and 293T DDX3KO/DDX3 cells infected with SeV for 12 h. (**e**) q-PCR analysis of *IFNB* mRNA in 293T, 293T DDX3KO, and 293T DDX3KO/DDX3 cells stimulated by 5’ppp-dsRNA transfection for 12 h. Data are presented as the mean ± SEM and are one representative of 3 independent experiments. Data were analyzed using two-way ANOVA with Sidak's (**a**-**c**) and Tukey's (**d**, **e**) post-test. *p < 0.05, **p < 0.001. ns, not significant.

These cells were transfected with 5′-triphosphate double-stranded RNA (5′ppp-dsRNA), a ligand for RIG-I or high molecular weight (HMW) poly (I:C), a ligand for MDA5 or infected with Sendai virus (SeV) or Newcastle disease virus (NDV). As predicted, *IFNB* expression was significantly induced by these stimuli in wild type (WT) cells (Fig. 1a). In DDX3 KO cells, these responses were markedly attenuated. To rule out the possibility of cell type specificity, we performed the above experiments in HeLa and HeLa DDX3 KO cells, and observed similar results (Supplementary Fig. 1c).

Next, we investigated the induction kinetics of *IFNB* gene by SeV in WT and DDX3 KO 293T (Fig. 1b) and HeLa cells (Supplementary Fig. 1d). DDX3 was required for efficient SeV-induced *IFNB* gene expression at all time points (Fig. 1b). Consistent with the attenuated IFN response, *SeV* RNA levels markedly increased in DDX3 KO 293T (Fig. 1c) and HeLa cells (Supplementary Fig. 1e).

To confirm the increased expression of *IFNB* mediated by DDX3 and rule out the possibility that attenuated *IFNB* induction occurred due to off-target effects of DDX3 gRNAs, we stably complemented the KO cells with WT Flag-DDX3. The reconstituted DDX3 expression was confirmed by Western blot analysis (Supplementary Fig. 1b). WT, DDX3 KO, and DDX3 KO/DDX3 293T cells were then infected with SeV (Fig. 1d) or stimulated with 5′ppp-dsRNA (Fig. 1e). *IFNB* gene expression induced by SeV and 5′ppp-dsRNA was restored to the same level as that in WT cells by complementation of DDX3. Altogether, these data confirm that DDX3 acts as a positive regulator in RLR-induced *IFNB* gene expression. Again, the complementation was confirmed using HeLa cells (Supplementary Fig. 1f, g).

### DDX3 regulates virus-induced signaling at the level of IRF-3

**Figure 2:**
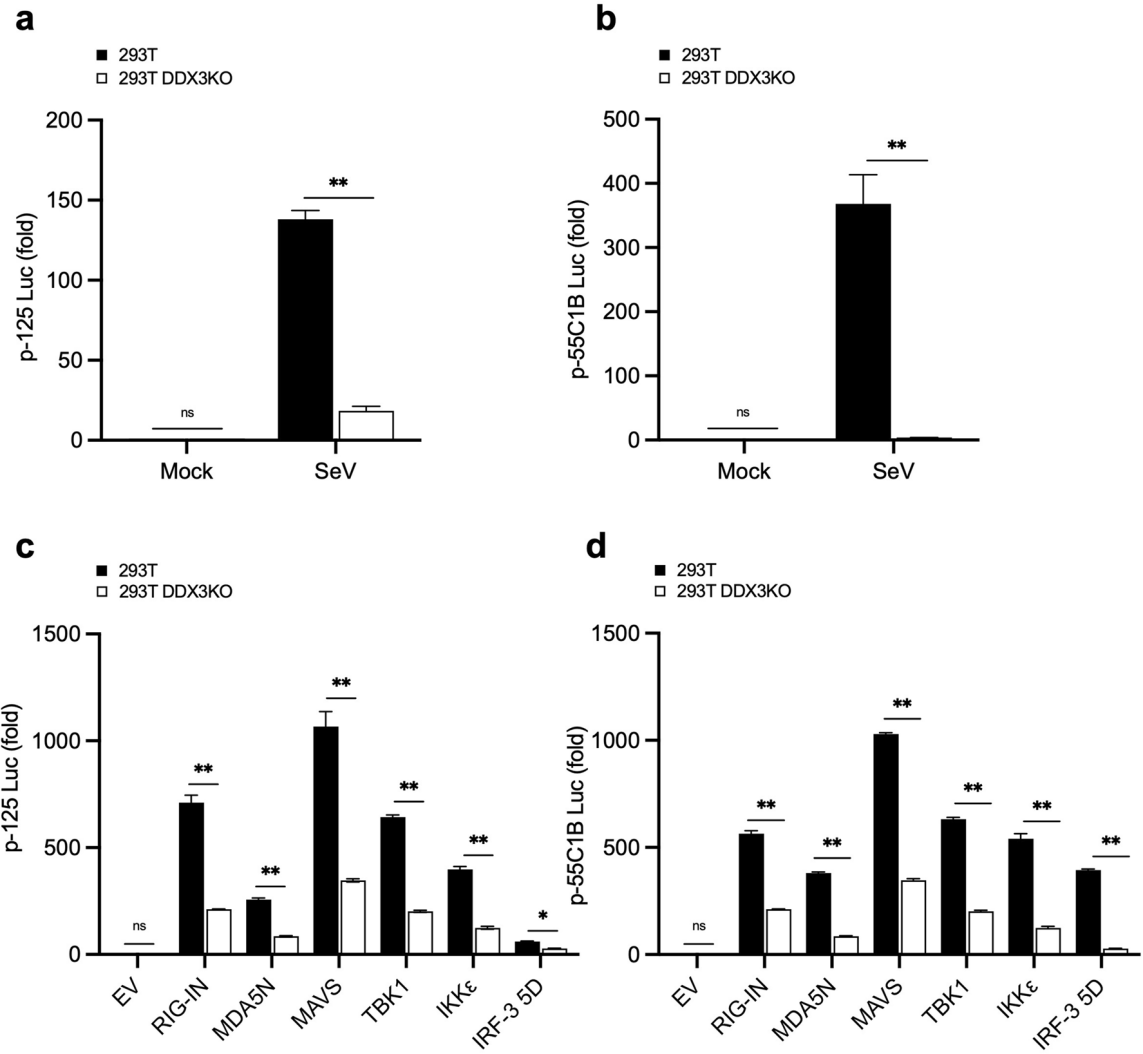
Effects of the absence of DDX3 on *IFNB* reporter gene expression stimulated by SeV or by expression of constitutive active signaling adaptors. 293T and 293T DDX3KO cells were transiently transfected with a reporter gene driven by *IFNB* promoter (p-125Luc, **a**) or reporter constructs containing repeated IRF-binding motifs (p-55C1BLuc, **b**) together with internal control (pRL-TK) plasmid for 24 h, followed by stimulation by infection with SeV for 12 h. Data represent relative firefly luciferase activity normalized to the internal control. 293T and 293T DDX3KO cells were transiently transfected with reporters (**c**: p-125Luc; **d**: p-55C1BLuc). Cells were simultaneously stimulated by co-transfection with expression vectors for RIG-IN, MDA5N, MAVS, TBK1, IKKɛ, or IRF-3 5D for 24 h. Cells were subjected to dual luciferase assay. Data are presented as relative firefly luciferase activity normalized to Renilla luciferase activity. Data are presented as the mean ± SEM and are one representative of 3 independent experiments. Data were analyzed using two-way ANOVA with Sidak's post-test (**a**–**d**). *p < 0.05, **p < 0.001. ns, not significant.

To ascertain the role of DDX3 in *IFNB* promoter regulation, we used a reporter gene controlled by the promoter region of *IFNB* (− 125 to + 19, p-125Luc) and that controlled by 8 repeats of the IRF binding site (p-55C1BLuc)^29^. These reporter genes were efficiently activated by SeV infection in 293T WT cells, but were markedly attenuated in 293T DDX3 KO cells (Fig. 2a, b). HeLa and HeLa DDX3 KO cells observed similar results (Supplementary Fig. 2a, b). These results are consistent with a previous study^26^ and suggest that DDX3 positively regulates the *IFNB* gene through IRF binding sites.

To further clarify the signaling steps in which DDX3 participates, we overexpressed a series of constitutive active signaling adaptors that bypass the viral trigger in 293T, 293T DDX3 KO (Fig. 2c, d), HeLa and HeLa DDX3 KO cells (Supplementary Fig. 2c). Overexpression of CARD of RIG-I and MDA5 directly relay signals to MAVS to trigger the downstream signaling^2^. Ectopic overexpression of MAVS, which results in its strong aggregation on mitochondria, also triggers the downstream signaling^30^. Transient overexpression of the regulatory protein kinases TBK1 and IKKε, which are responsible for IRF-3 phosphorylation, result in the phosphorylation of IRF-3 on Ser 386^31,32^, leading to *IFNB* gene activation. The IRF-3 5D mutant is constitutively phosphorylated IRF-3 on Ser 386 in human cells by an unknown kinase^31^ and mimics virus-induced *IFNB* gene induction. Interestingly, these constitutive active regulators activated p-125Luc and p-55C1BLuc reporters in a DDX3-dependent manner. These data suggest that DDX3 positively regulates the activation of *IFNB* at the level of IRF-3.

### DDX3 does not affect conventional innate signaling, including IRF-3 phosphorylation, dimerization, or nuclear translocation

**Figure 3:**
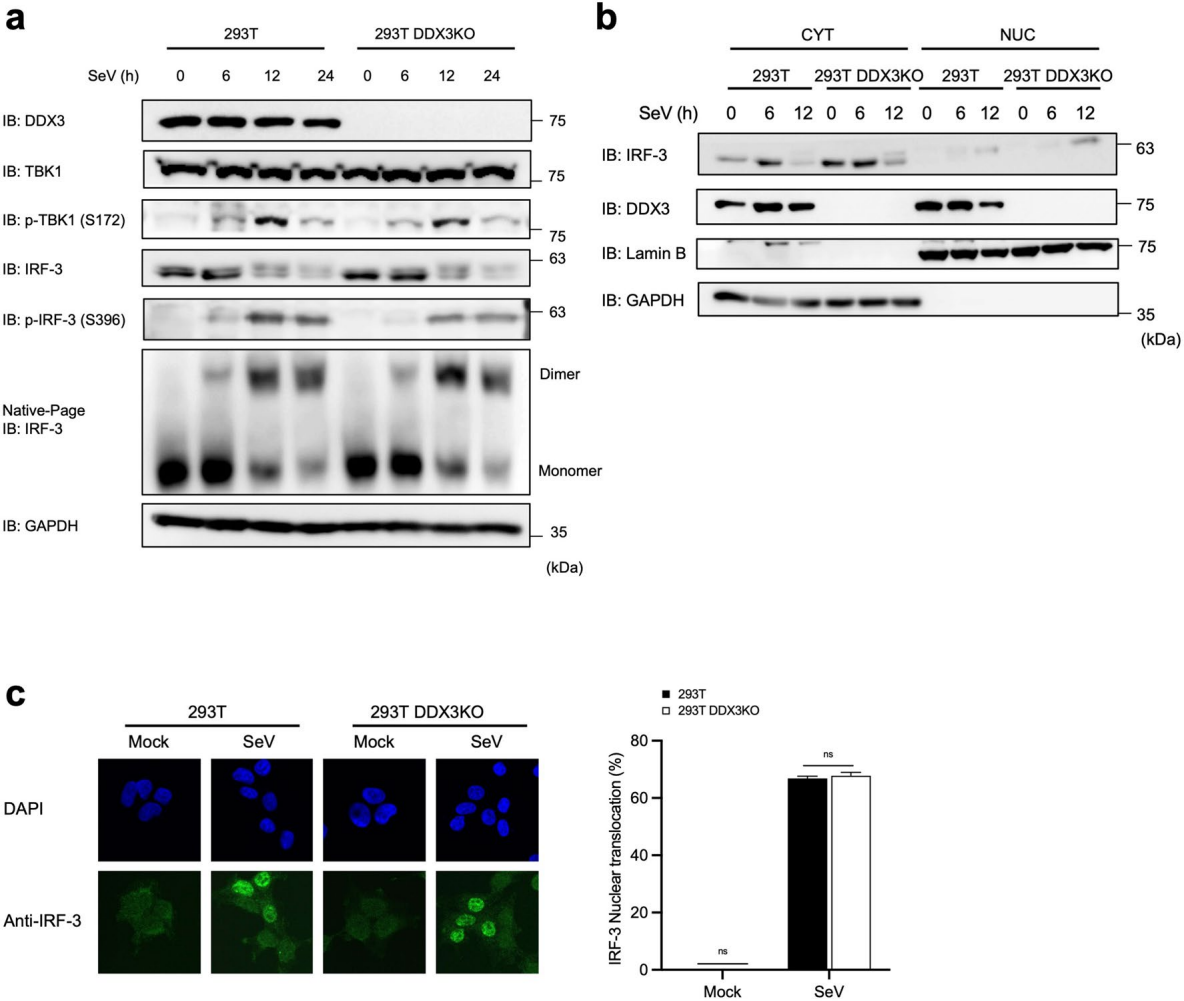
Analysis of different signaling events induced by SeV infection in 293T and 293T DDX3KO cells. (**a**) 293T and 293T DDX3KO cells were mock treated or infected with SeV for the indicated time. Total cell lysates were subjected to SDS-PAGE or native PAGE, followed by immunoblotting using the indicated antibodies. Full-length gels in Supplementary Information. (**b**) 293T and 293T DDX3KO cells were mock treated or infected with SeV for 12 h, and then subjected to subcellular fractionation. Cytoplasmic and nuclear extracts were examined by immunoblotting using the indicated antibodies. Full-length gels in Supplementary Information. (**c**) 293T and 293T DDX3KO cells were mock treated or infected with SeV for 12 h. Cells were fixed and stained for endogenous IRF-3 (green) and nuclear DNA was stained with DAPI (blue). Cells were observed by confocal microscopy and scored for nuclear translocation of IRF-3. Cells with nuclear IRF-3 were quantified as described in Methods. Data were analyzed using two-way ANOVA with Sidak's post-test. ns, not significant.

We examined the kinetics of TBK1 phosphorylation, IRF-3 phosphorylation, and IRF-3 homodimer formation (Fig. 3a) in virus-infected 293T and 293T DDX3 KO cells. Upon SeV infection, the total amount of TBK1 did not change; however, phospho TBK1 (S172) was detectable after 6 hpi and persisted thereafter. IRF-3 was detectable at 0 hpi and progressive mobility retardation in SDS gel was observed after 6 hpi, suggesting its phosphorylation at multiple sites. When the blot was probed with phospho-specific antibody (S396), IRF-3 phosphorylation was observed after 6 hpi and further increased thereafter. Native gel analysis revealed that IRF-3 dimer formation correlated with IRF-3 phosphorylation. Importantly, there was no notable difference in the phosphorylation of TBK1, IRF-3, or IRF-3 dimer formation in the presence or absence of DDX3.

Next, we examined IRF-3 nuclear translocation in SeV-infected cells by cell fractionation and immunofluorescence microscopy. Cell fractionation revealed that IRF-3 species with slow gel mobility were selectively translocated into the nucleus, suggesting phosphorylation-specific translocation in both WT and DDX3 KO cells (Fig. 3b). This observation was confirmed by staining with anti IRF-3 antibody (Fig. 3c). Counting of cells revealed that 66.9% and 67.8% of WT and DDX3 KO cells, respectively, exhibited nuclear translocation of IRF-3, suggesting that IRF-3 nuclear translocation is DDX3-independent.

Collectively, our study reveals that DDX3 does not affect the phosphorylation, dimerization, or nuclear translocation of IRF-3, but it may have some downstream effects after the activation of IRF-3.

### Physical association between DDX3 and IRF-3 requires the N-terminal DNA binding domain of IRF-3

**Figure 4:**
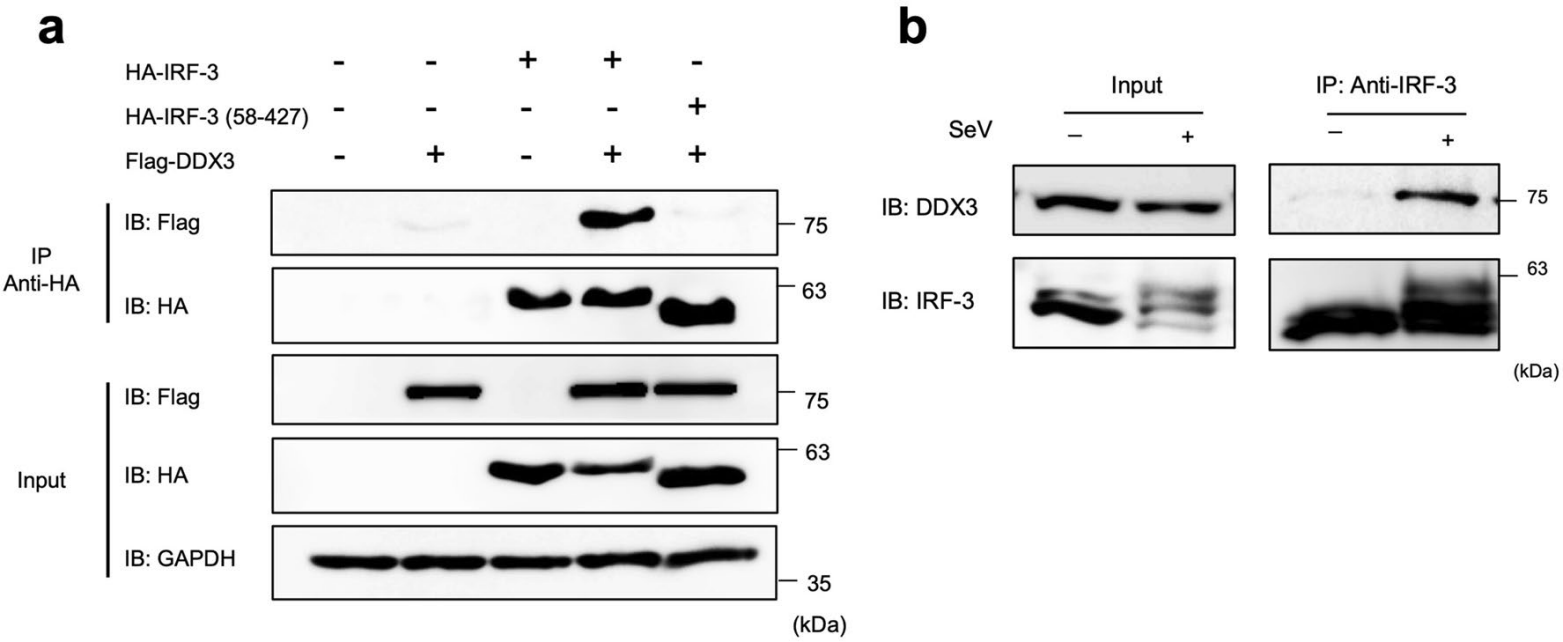
Physical interaction of IRF-3 and DDX3. (**a**) 293T cells were transfected with expression vectors for Flag-DDX3, HA-IRF-3 WT, or HA-IRF-3 (58-427) as indicated. Whole cell lysates were subjected to immunoblotting for Flag or HA epitope (input). Whole cell lysates were immunoprecipitated for HA epitope (IRF-3) and the precipitates were analyzed by immunoblotting for Flag or HA epitope (IP Anti HA). Data are representative of two independent experiments. Full-length gels in Supplementary Information. (**b**) Endogenous interaction between IRF-3 and DDX3 in 293T cells. 293T cells were mock treated or infected with SeV for 12 h. Whole cell lysates were immunoprecipitated by anti IRF-3 and the precipitates were analyzed by immunoblotting for IRF-3 or DDX3. Full-length gels in Supplementary Information.

Next, we examined the physical association of DDX3 with IRF-3. HA-IRF-3 and Flag-DDX3 were expressed in 293T cells, and co-immunoprecipitated. Consistent with previous reports, physical interaction between DDX3 and IRF-3 was confirmed (Fig. 4a). Endogenous interaction between DDX3 and IRF-3 was also confirmed (Fig. 4b). Truncated IRF-3 (58–427), devoid of its DNA-binding domain, did not associate with DDX3, indicating that the interaction intact DNA-binding domain of IRF-3 is required for the interaction.

### DDX3 is required for efficient IRF motif binding of the IRF-3 holocomplex

**Figure 5:**
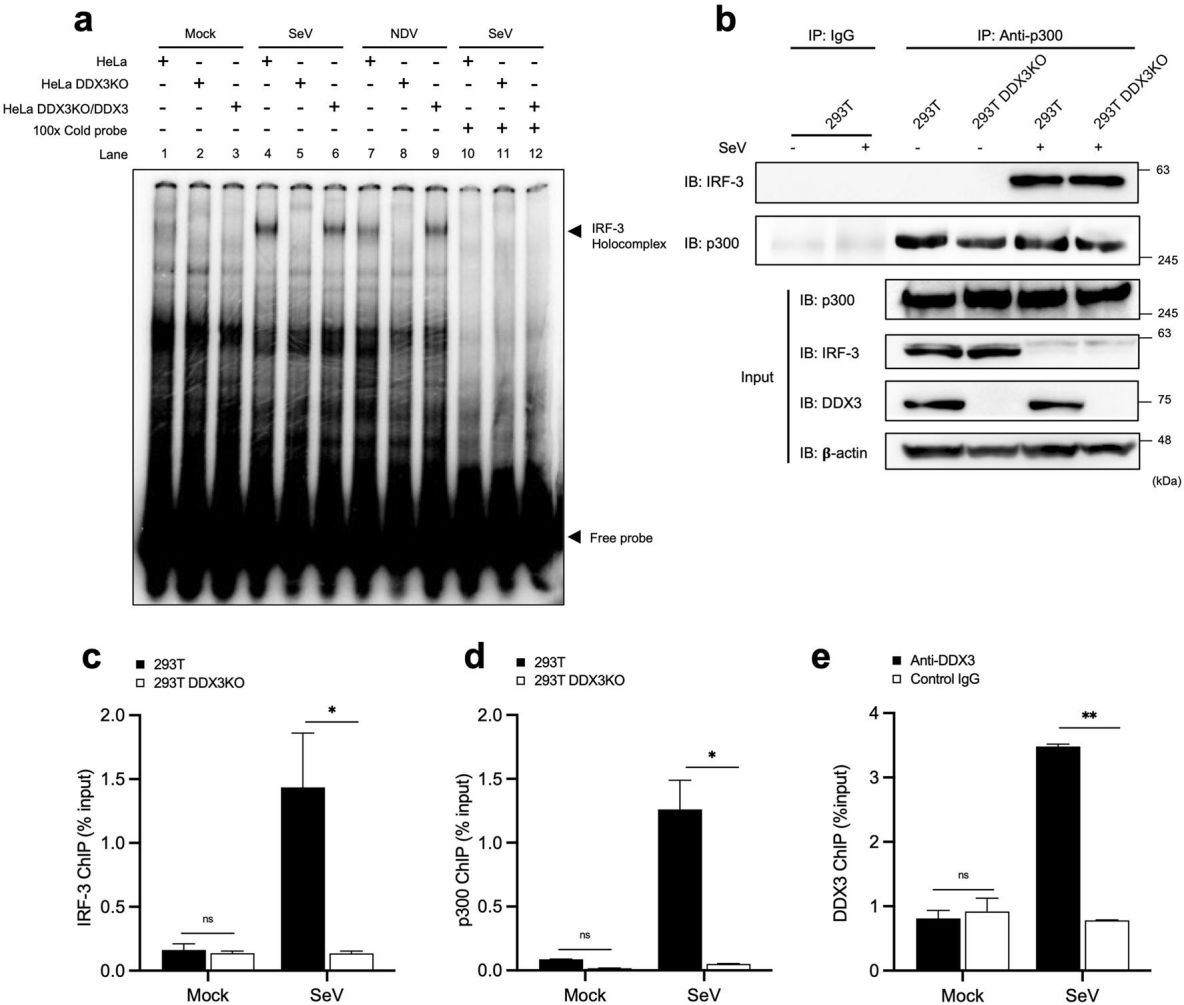
DDX3 is dispensable for the formation of a complex composed of IRF-3 and p300, but essential for its specific binding to the IRF motif DNA sequence. (**a**) HeLa, HeLa DDX3KO, or HeLa DDX3KO/DDX3 cells were transiently transfected with the expression vector for IRF-3 and mock treated or infected with SeV or NDV as indicated. Nuclear extract was prepared and subjected to EMSA with γ^32^P -labeled oligonucleotides containing the IRF-binding sequence. Unlabeled IRF-oligonucleotides (100-fold excess) were included to examine binding specificity (lanes 10–12). (**b**) 293T or 293T DDX3KO cells were infected with SeV for 12 h. Whole cell lysates were subjected to immunoblotting for p300, IRF-3, DDX3, or β-actin (input). Whole cell lysates were immunoprecipitated by control antibody (IgG) or anti-p300 and the precipitates were analyzed by immunoblotting for IRF-3 or p300. Full-length gels in Supplementary Information. (**c**) ChIP-qPCR assays of 293T and 293T DDX3KO cells stimulated by infection with SeV for 12 h. Chromatin was immunoprecipitated with anti IRF-3 antibody. (**d**) ChIP-qPCR assay as in (**c**) except with immunoprecipitation by anti p300 antibody. (**e**) ChIP-qPCR assay as in (**c**) but except with immunoprecipitation by control IgG or anti DDX3. Data are indicated as % of the DNA input. Data are presented as the mean ± SEM and are one representative of two independent experiments. Data were analyzed using two-way ANOVA with Sidak's post-test (**c**–**e**). *p < 0.05, **p < 0.001. ns, not significant.

The IRF-3 holocomplex composed of IRF-3 dimer and coactivator p300/CBP specifically binds to the IRF binding motif and p300/CBP is essential for the DNA binding^9,33^. We examined the IRF motif binding activity of the extract from WT and DDX3 KO (Fig. 5a). Conventional EMSA revealed that WT cell extract from virus-infected cells exhibited DNA/protein complex (IRF-3 holocompex) and the binding was abolished by competition of excess unlabeled probe DNA. However, the IRF-3 holocomplex was barely detectable in the similar extract from DDX3 KO cells. Of note, the holocomplex binding was restored in DDX3 KO cells complemented with the DDX3 expression vector (DDX3KO/DDX3). Moreover, the SeV infection-dependent association between IRF-3 and p300 was independent of DDX3 (Fig. 5b and Supplementary Fig. 3a). The above in vitro results prompted us to examine IRF-3 binding with the *IFNB* promoter in cells. We performed ChIP assay to detect binding of IRF-3 (Fig. 5c), p300 (Fig. 5d), and DDX3 (Fig. 5e) to the *IFNB* promoter. IRF-3, p300, and DDX3 associated with the *IFNB* promoter in SeV-infected WT cells, but the association was at a background level in DDX3 KO cells.

We performed the above experiments in HeLa and HeLa DDX3 KO cells and observed similar results (Supplementary Fig. 3b-d). Together, these results demonstrate that DDX3 contributes to the DNA binding activity of the IRF-3 holocomplex in vitro and in cells.

### ATP binding motif of DDX3 was required for efficient *IFNB* gene activation by virus infection

**Figure 6:**
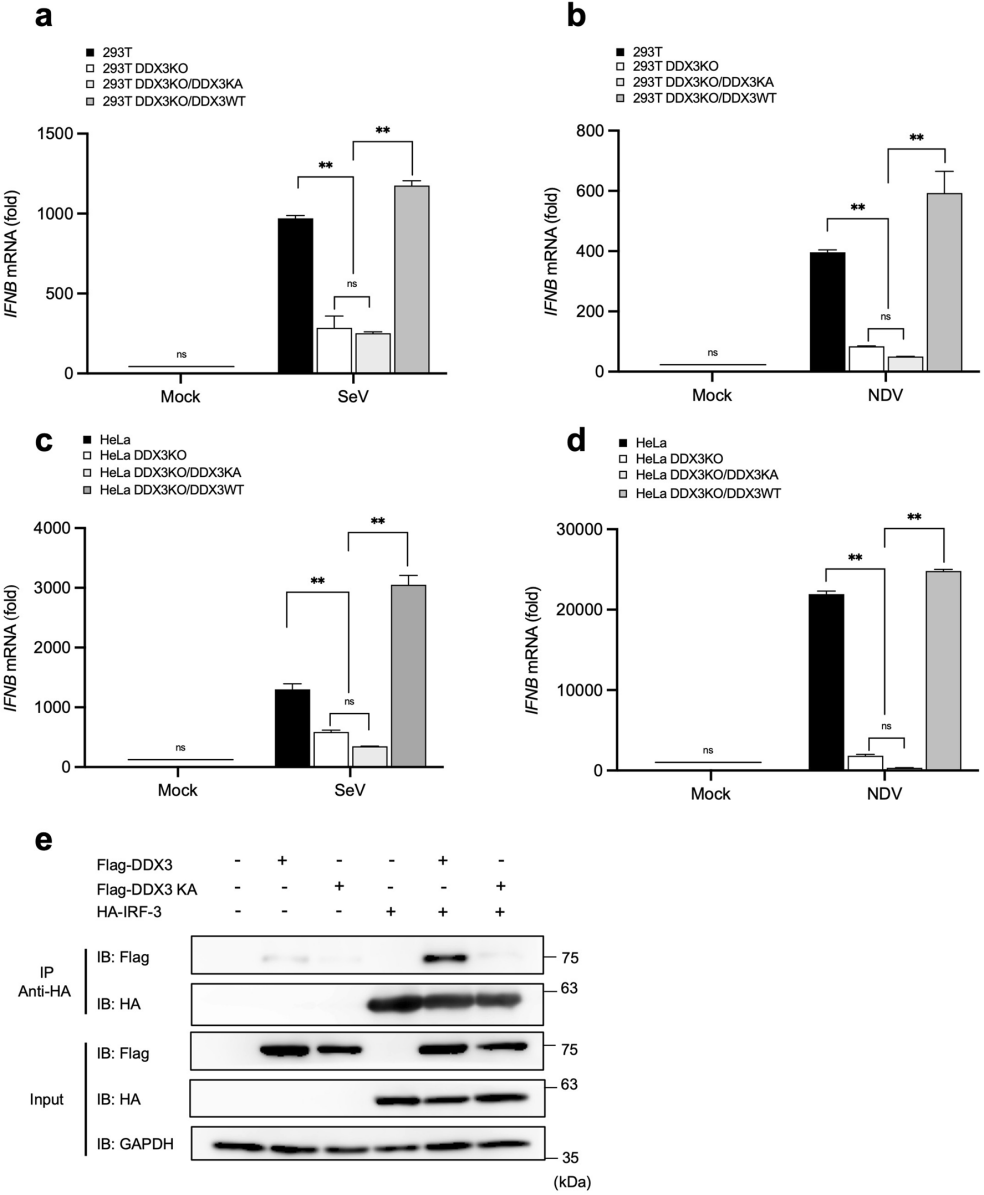
Failure of DDX3KA to complement DDX3 function to support *IFNB* gene expression and to interact with IRF-3 q-PCR analysis of *IFNB* mRNA in 293T, 293T DDX3KO, 293T DDX3KO/DDX3KA, and 293T DDX3KO/DDX3WT cells infected with SeV (**a**) and NDV (**b**) for 12 h. q-PCR analysis of *IFNB* mRNA in HeLa, HeLa DDX3KO, HeLa DDX3KO/DDX3KA, and HeLa DDX3KO/DDX3WT cells infected with SeV (**c**) and NDV (**d**) for 12 h. The results are presented as fold expression of *IFNB* mRNA to that of *GAPDH*. Data are presented as the mean ± SEM and are one representative of two independent experiments. Data were analyzed using two-way ANOVA with Tukey's post-test (**a**–**d**). **p < 0.001. ns, not significant. (**e**) 293T cells were transiently transfected with the expression vector for Flag-DDX3, Flag-DDX3KA, or HA-IRF-3 as indicated. Whole cell lysates were subjected to immunoblotting for Flag, HA, and GAPDH (input). Whole cell lysates were immunoprecipitated by anti HA and the precipitates were analyzed by immunoblotting for HA or Flag. Full-length gels in Supplementary Information.

Previous studies revealed that DDX3 mutant lacking ATPase activity promoted IFN-β induction by TBK1/IKKɛ overexpression^24,26^. We re-examined WT, DDX3 KO, and DDX3 KO stably expressing DDX3 K230A (KA) 293T cells (Fig. 6a, b) and HeLa cells (Fig. 6c, d) for *IFNB* gene activation. Unlike WT DDX3, DDX3 KA failed to complement the DDX3 deficiency in *IFNB* gene activation. Next, we examined the association of DDX3 KA with IRF-3, as shown in Fig. 4 (Fig. 6e). Efficient association of IRF-3 with DDX3 was observed, but not with DDX3 KA.

## Discussion

DDX3 has been implicated in carcinogenesis and human DDX3 syndrome; however, the underlying mechanism(s) for its physiological function are largely unknown. There have been several studies on the regulation of antiviral innate immunity by DDX3. Invading and replicating viral RNA is detected by sensor molecules termed RLR. The three members of RLR belong to the DExD/H-box protein superfamily of RNA helicases. Due to structural similarity, it was hypothesized that DDX3 functions in the sensing step of antiviral responses. However, the published reports are not consistent. We re-examined the function of DDX3 in antiviral immunity. Our results confirm that DDX3 positively regulates virus-induced type I IFN production (Figs. 1, 2). We utilized genome editing technology to avoid off-target effects of siRNA-mediated knockdown. We strictly confirmed that the loss of function of DDX3 by genome editing was complemented by ectopic expression of DDX3. Previous reports suggested that DDX3 facilitates the sensing of viral RNA or subsequent signaling steps. Our study, however, demonstrated that all the steps leading to the activation of *IFNB* gene were comparable in the presence or absence of DDX3 (Figs. 3, 5b), except that the IRF motif-DNA binding activity of the transcriptional factor complex composed of IRF-3 dimer and p300/CBP, was undetectable in the absence of DDX3 (Fig. 5a). Furthermore, chromatin immunoprecipitation demonstrated that IRF-3, p300, and DDX3 are specifically recruited to the *IFNB* gene promoter in SeV-infected cells, but not in uninfected cells (Fig. 5c–e). This notion is consistent with previous observation^26^. DDX3 is dispensable for the formation of the complex of IRF-3 dimer and p300/CBP (Fig. 5b). DDX3 has an intrinsic property to physically associate with IRF-3 (Fig. 4). We demonstrated that the DNA binding domain of IRF-3 (Fig. 4a) and ATP binding pocket of DDX3 (Fig. 6e) are involved in this association, and the latter is essential for transcriptional activation (Fig. 6a-d). Previous studies reported that the ATP binding pocket is dispensable for the transactivation^24,26^. This discrepancy is likely due to the use of reporter gene in contrast to our analysis of endogenous chromosomal *IFNB*. Although we do not have a precise explanation, the reporter gene strongly responded to the overexpression of DDX3 KA, a phenomenon not observed with chromosomal *IFNB* (Supplementary Fig. 4). The involvement of ATP hydrolysis by DDX3 in the transcriptional activation remains to be investigated. A similar scenario has also been described; Chao et al. (2016) have demonstrated that DDX3 acts as a transcriptional regulator by upregulating the promoter activity of the p21waf1/cip1 by interacting with the transcription factor Sp1. This study also provided evidence that DDX3 functioned on p21waf1/cip1 promoter through an ATPase-dependent but helicase-independent manner^34^.

**Figure 7:**
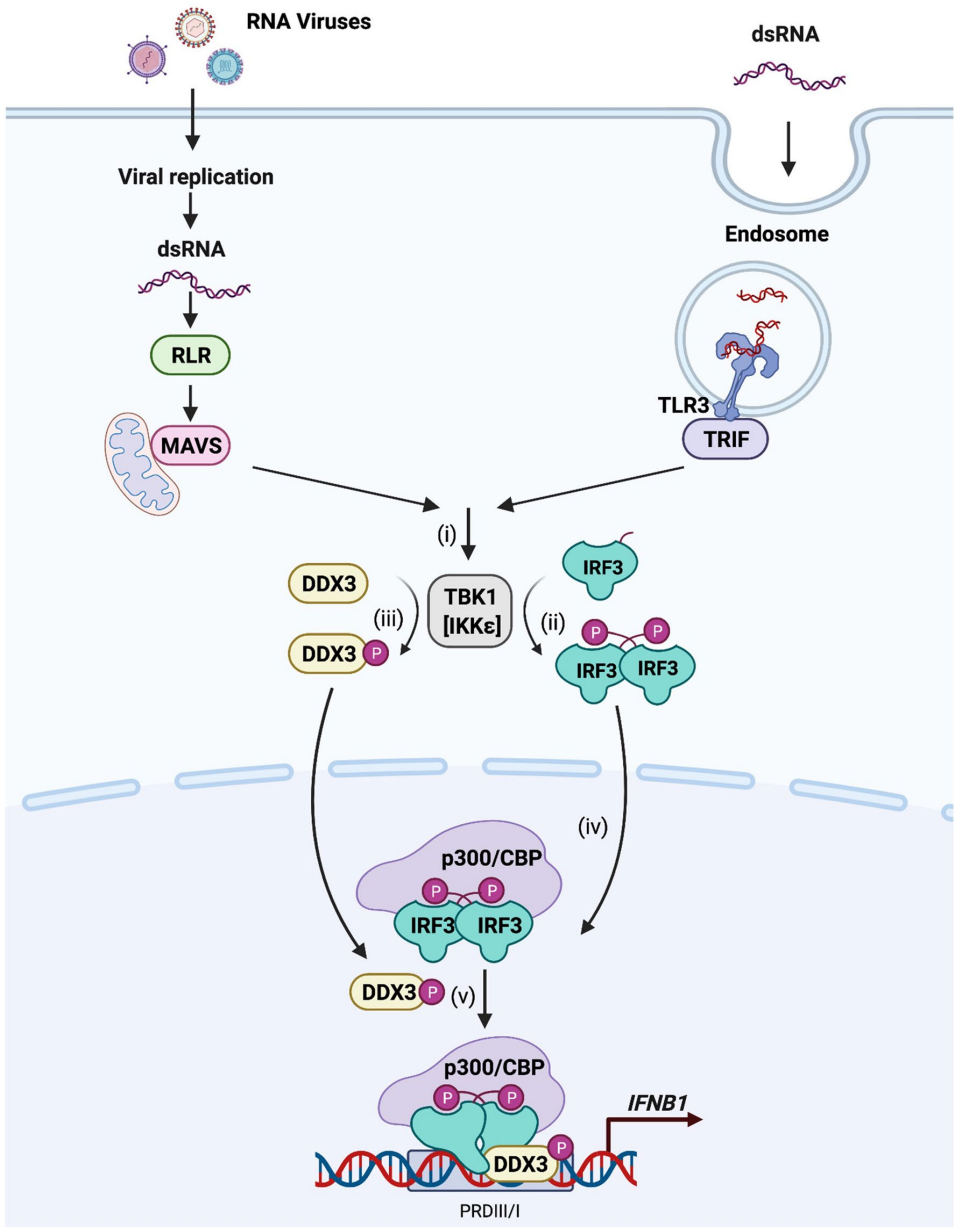
Proposed model for the functional role of DDX3 in RLR/TLR signaling. RLR/TLR3 senses viral dsRNA then transmits a signal to activate TBK1 (or IKKɛ,) (i). TBK1 interacts and phosphorylates IRF-3, leading IRF-3 to dimerize (ii); at the same time, TBK1 phosphorylates DDX3^25,26^(iii). Dimerized IRF-3 translocates to the nucleus, forming a complex with p300/CBP (vi). Phosphorylated DDX3 is recruited to the IRF-3/p300/CBP complex to confer its full binding activity to the *IFNB* promoter (v).

Based on the above, a model for virus-induced activation of type I IFN mediated by DDX3 was constructed (Fig. 7). Upon sensing viral dsRNA by RLR or TLR3, a signal is transmitted to activate TBK1 (or IKKε) (i). TBK1 phosphorylates IRF-3 at Ser 386 to induce its dimerization (ii). Previous studies have shown that DDX3 is directly phosphorylated by TBK1 and IKKε and that this phosphorylation mediates IRF-3 binding^25,26^ (iii). Dimerized IRF-3 translocates to the nucleus, where it forms a complex with p300 or CBP; however, in the absence of DDX3, this complex has little activity to recognize the target DNA (vi). Phosphorylated DDX3 is recruited to the IRF-3/p300/CBP complex to confer its full binding activity to the IRF motif sequence of the *IFNB* promoter (v). DDX3 plays an important role in guiding a transcription factor complex formed by antiviral signaling to the target gene promoter.

According to a previous study, DDX3Y increased *IFNB* gene expression in a similar way to DDX3X. In fibroblasts lacking both TBK1 and IKKε kinases, both DDX3X and DDX3Y increased the activity of a constitutively active IRF-7 mutant suggesting that TBK1/IKKε-mediated phosphorylation may not require the ability of DDX3X/Y to enhance IFN-β production. Furthermore, DDX3X was shown to enhance the activity of an NFκB reporter gene, suggesting its impact on innate immune responses may extend beyond the IRF-3/7 pathway^20^.

DDX3 belongs to a family of RNA helicases whose predicted function is to unwind dsRNA to ssRNA through ATP hydrolysis. The function of DDX3 revealed in the current study is novel and remotely related to that of RNA helicases. It is tempting to speculate that DDX3 regulates transcription factors other than IRF-3 to aid in their specific recognition of target genes. This may lead to an understanding of the versatile physiological functions of DDX3.

## Methods

### Cells and viruses

293T (#CRL-3216, ATCC) (female) and HeLa cells (#CCL-2.2, ATCC) (female) were cultured in Dulbecco’s modified Eagle medium (DMEM) (Nacalai Tesque) supplemented with 10% fetal bovine serum and penicillin–streptomycin (100 U/ml and 100 μg/ml, respectively). All cells were cultured in a humidified atmosphere with 5% CO_2_ at 37 °C.

DDX3 knockout 293T and HeLa cells were generated by transient transfection of the pSpCas9(BB)-2A-GFP (PX458) vector containing guide RNA (sgRNA: sg-DDX3 forward 5′-caccGTGGCAGTGGAAAATGCGCT-3′ sg-DDX3 reverse 5′-aaacAGCGCATTTTCCACTGCCAC-3′). Cells were subjected to sorting by green fluorescent protein expression levels using the SH800 cell sorter (Sony), followed by limiting dilution in 96-well culture plates to obtain single clones. Deletion of the DDX3 gene was examined by genome sequencing and Western blotting. Western blot analysis of DDX3 expression in 293T/HeLa DDX3 KO cells compared with WT cells using an antibody directed against the N- and C-terminus of DDX3 (Supplementary Fig. 1a).

The PCR primers used for genomic DNA are as follows: DDX3 DNA forward; 5′cagtagccgggcagaagtc-3′ and DDX3 DNA reverse 5′-aatacagcgggccgagac-3′.

To complement DDX3 expression in DDX3 KO cells, lentiviral particles were produced by introducing plasmids pCAG-HIVgp, pVSV-G-RSV-ReV, and pCSII-CMV-MCS-IRES2-Bsd, encoding DDX3 or DDX3 KA, into 293T and HeLa cells. Transfection was performed using polyethylenimine (PEI) (Polysciences) for 293T cells and Lipofectamine 2000 (Invitrogen) for HeLa cells in 10-cm dishes. Media were changed at 6 h post-transfection (hpt), and viral supernatants were collected at 48 and 72 hpt and filtered (0.45 µm). Polybrene (10 µg/ml) was added to the filtered supernatants and used to infect cells. The infected cell lines were selected with blasticidin (10 µg/ml). Colonies were picked and examined for the expression of DDX3 by Western blotting.

Virus infection was performed as previously described^35^.

### Construction of the DDX3 and mutant expression plasmids

Expression plasmid of DDX3 was constructed by RT-PCR amplification of cDNAs from 293T cells with primers encoding the Flag tag at its N-terminus. The PCR products were subcloned into pEF-Bos^31^. pEF-Flag DDX3 K230A was generated by site-directed mutagenesis of pEF-Flag DDX3 using KOD-plus site-directed mutagenesis (Toyobo). pEF-Flag DDX3 K230A mutant was confirmed by sequencing. IRF-3 and IRF-3 (58-427) expression plasmids were described previously^7^. Reporter constructs p-125Luc and p-55C1BLuc were described previously^29^.

### Cell stimulation by RNA transfection and by transfection of expression vectors

Poly(I:C) and 5′ppp-dsRNA were transfected for 12 h at a concentration of 1 μg/ml using PEI for 293T cells and Lipofectamine 2000 for HeLa cells. For transient protein expression in 293T and HeLa cells, expression plasmids were transfected using PEI and Lipofectamine LTX, respectively.

### Reagents and antibodies

HMW Poly (I:C) was purchased from GE Healthcare. Mouse monoclonal anti-Flag M2 (F1804) was purchased from Sigma-Aldrich. Mouse monoclonal anti-β-actin (sc-47778) and goat polyclonal anti-Lamin B (M-20), mouse anti-DDX3 (C4), and mouse monoclonal anti-GAPDH (0411) were purchased from Santa Cruz Biotechnology. Purified rabbit anti-DDX3 (NB200-196) was purchased from Novus Biologicals. Mouse monoclonal anti-HA.11 (901502) and purified mouse anti-IRF-3 antibody (Go-ChIP) were purchased from BioLegend. Rabbit monoclonal anti-TBK1 (ab40676) was purchased from Abcam. Rabbit monoclonal anti-phospho TBK1 (Ser172) (5483), rabbit monoclonal anti-IRF-3 (Ser396) (D6O1M), and rabbit monoclonal anti-p300 (D8Z4E) were purchased from Cell Signaling Technology. Mouse monoclonal anti-IRF-3 (CBX00167) was purchased from Cosmobio.

### RNA isolation and real-time qPCR

Total RNA was extracted from cells using TRIzol reagent. cDNA was generated using ReverTra Ace® qPCR RT Master Mix with gDNA Remover (TOYOBO). Real-time qPCR was performed on the Step One plus real-time PCR system (Applied Biosystems) using THUNDERBIRD SYBR qPCR Mix (TOYOBO). Sequences of the primers used in real-time PCR are listed in Supplementary Table 1.

### Subcellular fractionation

Cells collected from 6-well plates were suspended in 25 μl of 0.1% NP-40 in PBS (supplemented with protease inhibitors cocktail), and then centrifuged at 4500*g* for 5 min at 4 °C. The supernatant was collected as the cytoplasmic fraction. Pellets were washed at least 5 times with 500 μl of 0.1% NP-40 in PBS. Nuclear pellets were then lysed with 20 μl of lysis buffer (20 mM Tris–HCl pH 7.5, 150 mM NaCl, and 1% NP-40 supplemented with protease inhibitors cocktail), and then sonicated briefly 3 times before centrifugation at 15,000*g* for 10 min at 4 °C. The supernatant was kept as the nuclear fraction.

### Immunoblotting and immunoprecipitation

293T cells were washed with PBS. Cells were lysed with lysis buffer. Cell extracts were centrifuged at 15,000*g* for 10 min at 4 °C, and supernatants were collected as protein extracts, re-suspended in 2X SDS sample buffer before boiling and electrophoresis.

For immunoprecipitation, protein extracts were incubated with a mixture of 1 μg of antibody overnight at 4 °C and then Dynabeads protein G (Thermo Fisher) was added and incubate for 4 h. The immunocomplexes were washed 3 times in lysis buffer, followed by denaturation in 2X SDS sample buffer. Denatured proteins were resolved by SDS-PAGE. Proteins on the membranes were visualized on the LAS-4000 instrument (Fujifilm) by chemiluminescence.

### Native PAGE

To detect IRF-3 dimerization, native PAGE was performed as described previously^31,35^. Briefly, a 7.5% polyacrylamide gel was pre-run with the running buffer (25 mM Tris–HCl pH 8.4, 192 mM glycine) in the presence or absence of 0.2% sodium deoxycholate (DOC) in the upper (−) and lower (+) chamber buffers, respectively, at 40 mA for 30 min. Protein extracts were mixed with 5X Native PAGE sample buffer (312.5 mM Tris–HCl pH 6.8, 50% glycerol, and 0.01% BPB). The samples were electrophoresed at 26 mA for 30 min at room temperature. Proteins were transferred onto PDVF membranes and detected by immunoblotting.

### Immunostaining

293T cells were cultured in 8-well chamber plates (Ibidi) and either left untreated or infected with SeV for 12 h. Cells were fixed with 4% paraformaldehyde (PFA), permeabilized with 0.25% Triton X-100 in PBS, and treated with 1% bovine serum albumin (BSA) in PBST for 30 min at room temperature before incubation with the primary antibody at 4 °C overnight. The secondary antibody was applied and incubated with the samples for 1 h at room temperature. Cells were counterstained with DAPI and then observed under a confocal microscope (TCS-SP8-Leica Microsystems). Nuclear localization of IRF-3 was analyzed with Keyence BZ-X700 microscope and scored with Keyence Analyzer software. More than 500 cells were counted 3 times for each sample.

### Luciferase reporter assay

293T cells were seeded in 24-well plates at 1 × 10^5^ cells per well in DMEM medium with 10% FBS overnight, and transfected with RIG-IN, MDA5N, MAVS, TBK1, IKKε, IRF-3 5D expression plasmids (200 ng) and p-125 Luc (200 ng) or p-55C1B Luc (200 ng) together with pRL-TK-Renilla Luc (10 ng). SeV infection was conducted at 24 hpt. At 12 hpi, cells were lysed with passive lysis buffer (Promega), and firefly luciferase and Renilla luciferase activities were measured using a dual-luciferase assay kit (Promega).

### Electrophoretic mobility shift assay (EMSA)

Nuclear extracts (10 µg) and 0.1 pmol of γ^32^P-labelled ISRE probe were incubated for 20 min at room temperature in 10 µl of binding mixture (20 mM HEPES pH 7.5, 100 mM NaCl, 1 mM DTT, 1 mM EDTA, 1% NP-40, 5% glycerol, and 0.2 µg of herring sperm DNA). The DNA–protein complexes were resolved by electrophoresis on 4% acrylamide gels in 1X TBE at 160 V for 1 h at room temperature. The oligonucleotide for ISRE probe is: 5′GAGAGGGAAACCGAAACTGAATTAGCTTTCAGTTTCGGTTTCCCTCT-3′ (the ISRE is underlined).

### Chromatin immunoprecipitation

293T cells were cultured in 15-cm dishes and either left untreated or infected with SeV for 12 h before fixation with 1% formaldehyde, followed by quenching using 125 mM glycine. Cell extracts were prepared with lysis buffer and then sonicated using a M220 focused-ultrasonicator (Covaris). After sonication, samples were centrifuged to remove insoluble debris, supernatants were collected, and 5% of each sample was used to measure chromatin input. The rest of the sample was diluted in ChIP dilution buffer (20 mM Tris–HCl, pH 7.5, 1 mM EDTA, 0.5 mM EGTA, 500 mM NaCl, 1% Triton-X-100, 0.1% SDS, and 0.1% DOC). Antibodies were conjugated to Dynabeads protein G (Thermo Fisher) by pre-incubation with 0.5% BSA for 6 h. The beads were incubated with lysate at 4 °C for overnight and washed 5 times with washing buffer (50 mM HEPES–KOH pH 7.4, 500 mM LiCl, 1 mM EDTA, 1% NP40, and 0.7% DOC). To elute the DNA, beads were shaken with 100 μl of elution buffer (50 mM Tris–HCl, pH 8, 10 mM EDTA, and 1% SDS) at 65 °C for 20 min. The immune complexes were reverse cross-linked at 65 °C overnight. After treatment with RNase A and proteinase K, DNA was purified by the Monarch PCR & DNA Cleanup Kit (NEW ENGLAND BioLabs). Real-time PCR analyses were performed using the recovered DNA. The primers of the *IFNB* promoter were: 5′-GAAAGGGAGAAGTGAAAGTGG-3′ (sense), 5′-AAGGCTTCGAAAGGTTGCAG-3′ (anti-sense).

### Statistical analysis

Statistical analysis was performed using Prism software (version 9). Two-way analysis of variance (ANOVA) was used for multiple comparison (*P < 0.05, **P < 0.001, ***P < 0.0001, ns, not significant).

## Supplementary Information

Supplementary Information.

## Data Availability

All data generated and analyzed during the study is included in the published article and Supplementary Information. The datasets used and/or analyzed during the current study are available from the corresponding author on reasonable request.
