# Supplementary material for: The RNA helicase DDX3 promotes IFNB transcription via enhancing IRF-3/p300 holocomplex binding to the IFNB promoter
Source: Sci Rep. 2022 Mar 10;12:3967. doi: 10.1038/s41598-022-07876-z (PMC8913847; doi:10.1038/s41598-022-07876-z)
Supplement: Supplementary file 1 — Supplementary Information. [file 41598_2022_7876_MOESM1_ESM.pdf]

Supplementary information

The RNA helicase DDX3 promotes *IFNB* transcription via enhancing IRF-3/p300 holocomplex binding to the *IFNB* promoter

Wilaiporn Saikruang[1,2], Lena Ang Yan Ping[1,2], Hiroto Abe[1,2], Dacquin M. Kasumba[1,2], Hiroki Kato[2,3], and Takashi Fujita[1,2,*]

[1] Division of Integrated Life Science, Graduate School of Biostudies, Kyoto University, Kyoto, 606-8501, Japan

[2] Laboratory of Regulatory Information, Institute for Frontier Life and Medical Sciences, Kyoto University, Kyoto, 606-8507, Japan

[3] Institute for Cardiovascular Immunology, University Hospital Bonn, University of Bonn, Bonn, 53127, Germany

*Corresponding Author:

Takashi Fujita (PhD)

Laboratory of Regulatory Information,

Institute for Frontier Life and Medical Sciences, Kyoto University,

53 Kawahara-cho, Shogoin, Sakyo-ku, Kyoto 606-8507, Japan

Tel/Fax: +81-75-751-4031

E-mail: fujita.takashi.86e@st.kyoto-u.ac.jp

Supplemental Figure S1

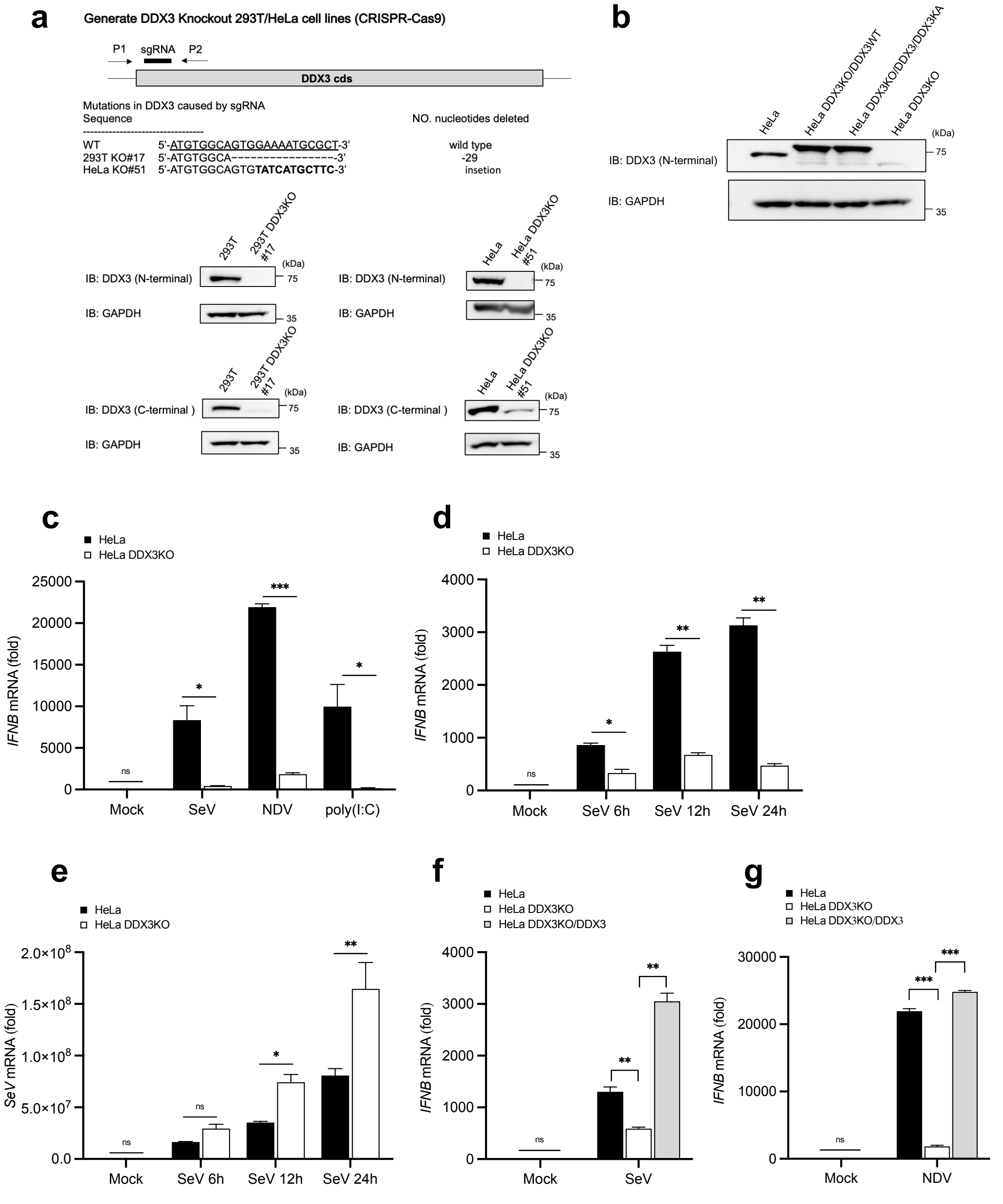

Supplementary Figure S1. (**a**) Schematic illustration of CRISPR/CAS9-mediated deletion of DDX3 and guide RNA. Sequences of WT and deleted alleles are shown; gRNA target sites are in black. The deletion junction was verified by sequencing. Western blot analysis of DDX3 expression in 293T/HeLa DDX3 KO cells compared with WT cells using an antibody directed against the N- and C-terminus of DDX3. GAPDH was used as a loading control. Full-length gels in Supplementary Information. (**b**) Detection of DDX3 protein by immunoblotting in HeLa, HeLa DDX3KO/DDX3WT, HeLa DDX3KO/DDX3KA, and HeLa DDX3KO cells. Full-length gels in Supplementary Information. (**c**) *IFNB* gene expression in

HeLa and HeLa DDX3KO cells. Cells were stimulated with infection by SeV, NDV (12 h) or transfection with poly I:C (HMW, 12 h) and *IFNB* mRNA was quantified. (**d**) q-PCR analysis of *IFNB* mRNA in HeLa and HeLa DDX3KO cells infected with SeV for indicated times. (**e**) q-PCR analysis of *SeV* RNA in HeLa and HeLa DDX3KO cells infected with SeV for indicated times. q-PCR analysis of *IFNB* mRNA in HeLa, HeLa DDX3KO, and HeLa DDX3KO/DDX3 cells infected with SeV (**f**) and NDV (**g**) for 12 h. The results were presented as fold expression of *IFNB* mRNA to that of *GAPDH* mRNA. Data are presented as the mean ± SEM and are one representative of 3 independent experiments. Data were analyzed using two-way ANOVA with Sidak's post-test (**c-g**). *$p < 0.05$, **$p < 0.001$, ***$p < 0.0001$. ns, not significant.

Supplemental Figure S2

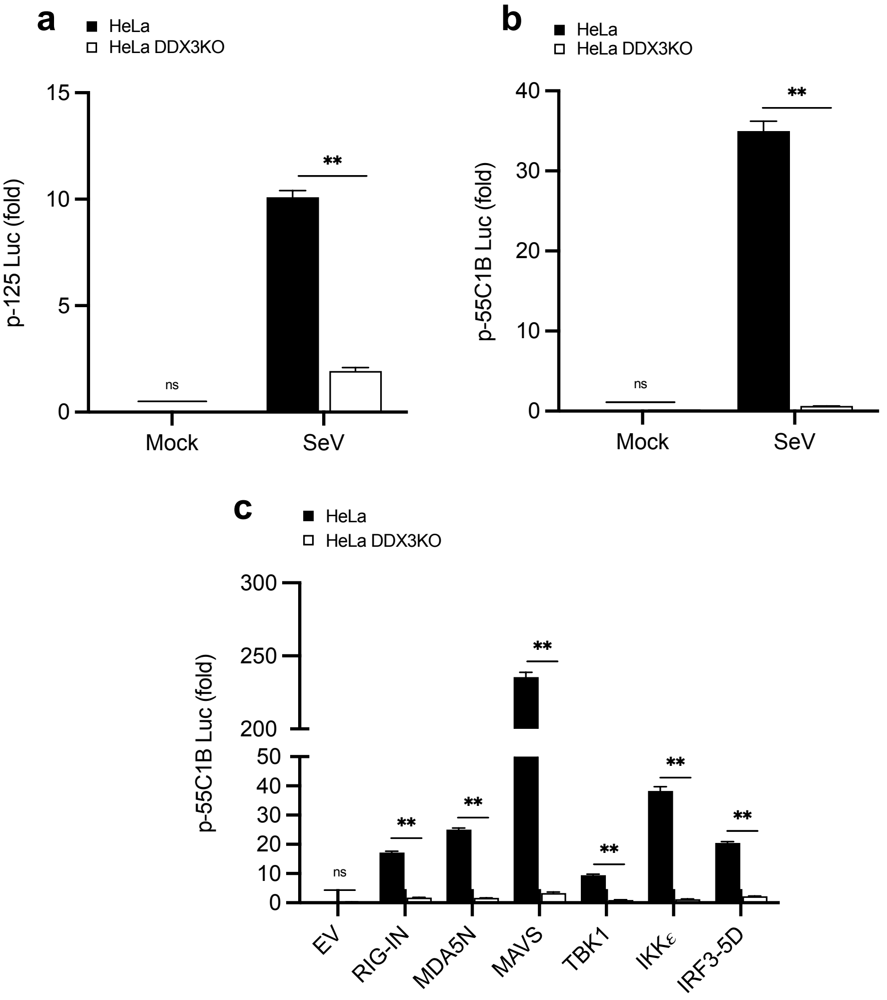

Supplementary Figure S2. Effects of the absence of DDX3 on *IFNB* reporter gene expression stimulated by SeV or by expression of constitutive active signaling adaptors. HeLa and HeLa DDX3KO cells were transiently transfected with a reporter gene driven by *IFNB* promoter (p-125Luc, **a**) or reporter constructs containing repeated IRF-binding motifs (p-55C1BLuc, **b**) together with internal control (pRL-TK) plasmid for 24 h, followed by stimulation by infection with SeV for 12 h. Data represent relative firefly luciferase activity normalized to the internal control. HeLa and HeLa DDX3KO cells were transiently transfected with reporters (**c**: p-55C1BLuc). Cells were simultaneously stimulated by co-transfection with expression vectors for RIG-IN, MDA5N, MAVS, TBK1, IKKε, or IRF-3-5D for 24 h. Cells were subjected to dual luciferase assay. Data are presented as relative firefly luciferase activity normalized to Renilla luciferase activity. Data are presented as the mean ± SEM and are one representative of 2 independent experiments. Data were analyzed using two-way ANOVA with Sidak's post-test (**a-c**). **p < 0.001. ns, not significant.

Supplemental Figure S3

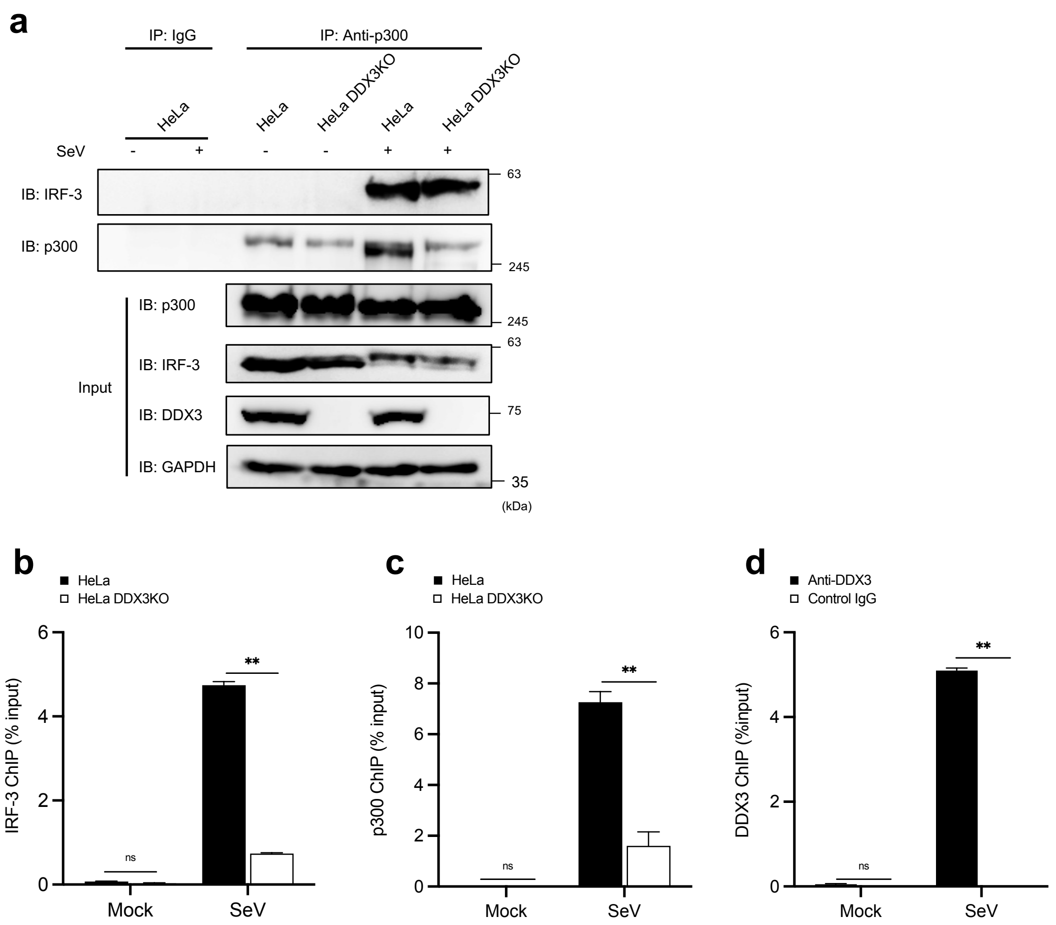

Supplementary Figure S3. (**a**) HeLa or HeLa DDX3KO cells were infected with SeV for 12 h. Whole cell lysates were subjected to immunoblotting for p300, IRF-3, DDX3, or GAPDH (input). Whole cell lysates were immunoprecipitated by control antibody (IgG) or anti-p300 and the precipitates were analyzed by immunoblotting for IRF-3 or p300. Full-length gels in Supplementary Information. (**b**) ChIP-qPCR assays of HeLa and HeLa DDX3KO cells stimulated by infection with SeV for 12 h. Chromatin was immunoprecipitated with anti IRF-3 antibody. (**c**) ChIP-qPCR assay as in (**b**) except with immunoprecipitation by anti p300 antibody. (**d**) ChIP-qPCR assay as in (**b**) but except with immunoprecipitation by control IgG or anti DDX3. Data are indicated as % of the DNA input. Data are presented as the mean ± SEM and are one representative of two independent experiments. Data were analyzed using two-way ANOVA with Sidak's post-test (**b-d**). $**p < 0.001$. ns, not significant.

Supplemental Figure S4

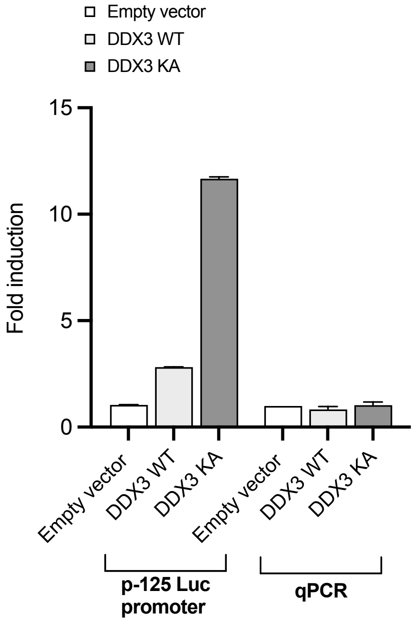

Supplementary Figure S4. Comparison of the effect of transiently overexpressed DDX3 WT and DDX3 KA plasmids on *IFNB* reporter gene expression (p-125Luc) and qPCR for endogenous *IFNB* in DDX3 KO 293T cells. Cells were transfected with empty vector, expression vectors for DDX3 WT and DDX3 KA for 24 h. Cells were subjected to dual luciferase and qPCR analyses. Luciferase activities are presented as relative firefly luciferase activity normalized to Renilla luciferase activity. qPCR results are normalized to GAPDH expression. Data are presented as the mean ± SEM and are one representative of two independent experiments.

Western blot original

Fig. 3a

IB: DDX3

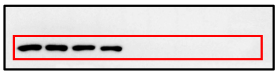

IB: TBK1

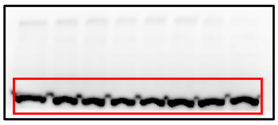

IB: p-TBK1 (S172)

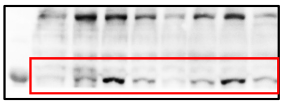

IB: IRF-3

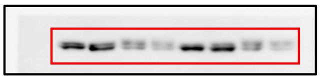

IB: p-IRF-3 (S396)

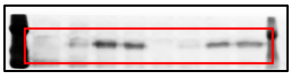

Native-Page
IB: IRF-3

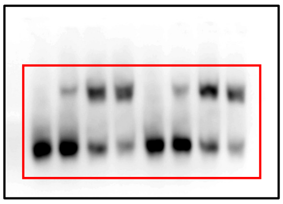

IB: GAPDH

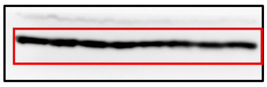

Fig. 3b

IB: IRF-3

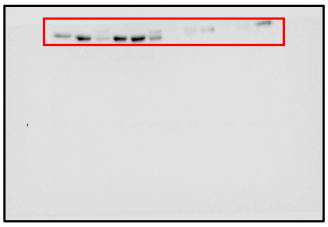

IB: DDX3

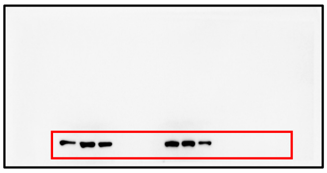

IB: Lamin B

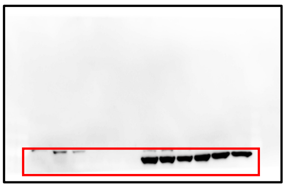

IB: GAPDH

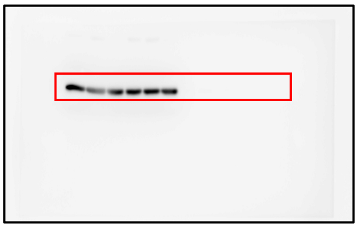

Western blot original

Fig. 4a

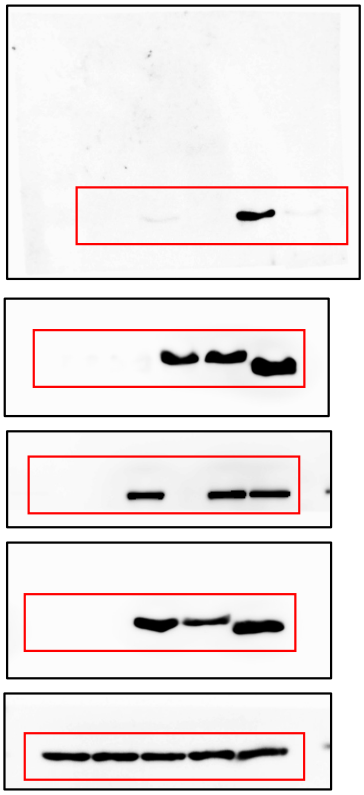

IB: Flag

IB: HA

IB: Flag

IB: HA

IB: GAPDH

Fig. 4b

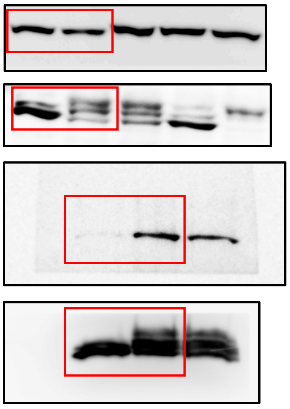

IB: DDX3

IB: IRF-3

IB: DDX3

IB: IRF-3

Fig. 5b

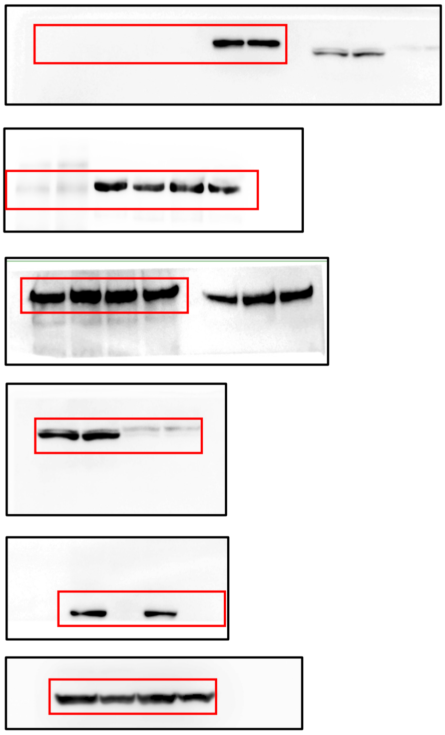

IB: IRF-3

IB: p300

IB: p300

IB: IRF-3

IB: DDX3

IB: β-actin

Western blot original

Fig. 6e

IB: Flag

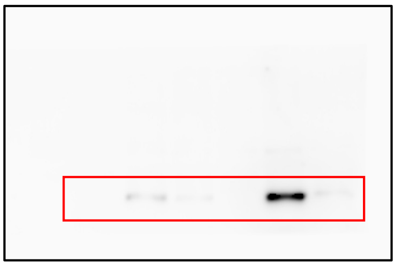

IB: HA

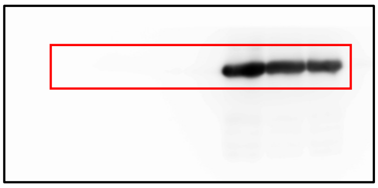

IB: Flag

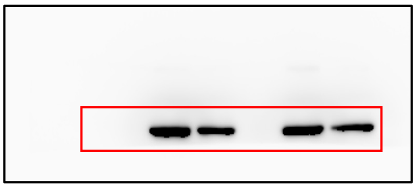

IB: HA

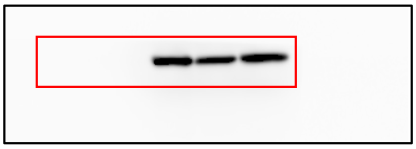

IB: GAPDH

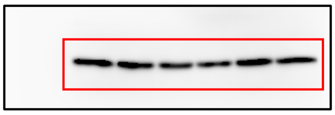

Fig. S1a

293T

IB: DDX3 ((N-terminal)

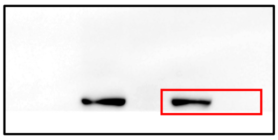

IB: DDX3 (C-terminal)

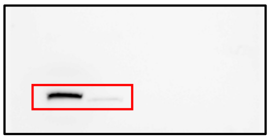

IB: GAPDH

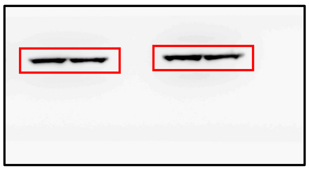

HeLa

IB: DDX3 ((N-terminal)

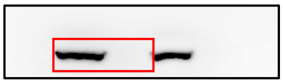

IB: GAPDH

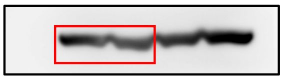

IB: DDX3 (C-terminal)

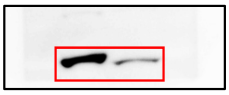

IB: GAPDH

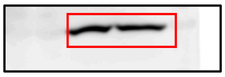

Fig. S1b

IB: DDX3

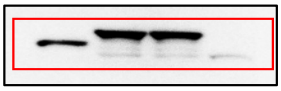

IB: GAPDH

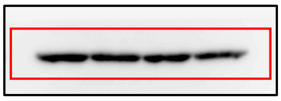

Western blot original

Fig. S3a

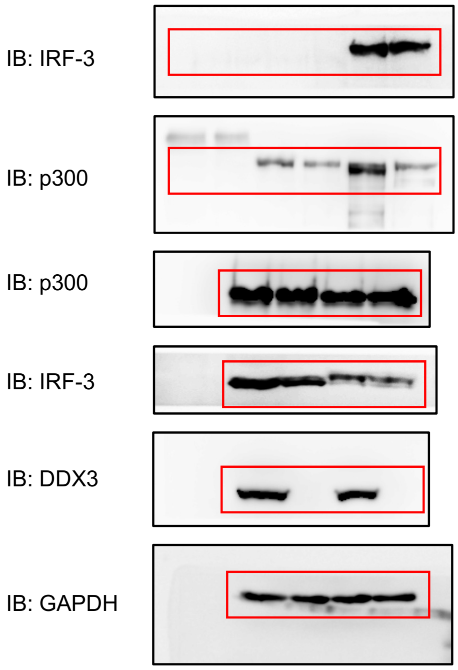

Supplementary Figure S2. Full scans of immunoblots. The boxed regions are presented in the indicated figures.

Supplementary Table 1. Primer sequences for PCR

Primer	Sequences
human *IFNB*	Forward: AGCTGCAGCAGTTCCAGAAG
	Reverse: AGTCTCATTCCAGCCAGTGC
human *GAPDH*	Forward: CTGCACCACCAACTGCTTAG
	Reverse: GTCTTCTGGGTGGCAGTGA
SeV (Cantell)	Forward:5'-GACGCGAGTTATGTGTTTGC-3'
	Reverse: 5'-TTCCACGCTCTCTTGGATCT-3'
